# Case Report: Vincristine extravasation managed with amniotic membrane graft in a pediatric patient with Wilms tumor

**DOI:** 10.3389/fped.2026.1926830

**Published:** 2026-08-19

**Authors:** Fatma Betul Ozen, Ayse Oz, Sultan Aydin

**Affiliations:** Department of Pediatric Hematology and Oncology, Antalya Training and Research Hospital, Antalya, Türkiye

**Keywords:** amniotic membrane, pediatric oncology, vincristine extravasation, Wilms tumor, wound healing

## Abstract

Vincristine is a commonly used chemotherapeutic agent in pediatric oncology and, because of its vesicant properties, may cause severe tissue injury in cases of extravasation. We present a pediatric case in which tissue damage developing after vincristine extravasation during treatment for Wilms tumor was managed with an amniotic membrane graft following clinical progression despite standard conservative measures. A 2 year eight month old boy developed vincristine extravasation from a peripheral intravenous line on the dorsolateral aspect of the left foot during week 24 of carboplatin–vincristine chemotherapy for Stage II Wilms tumor. The progression of tissue injury despite initial conservative management suggested that amniotic membrane grafting may represent a useful alternative therapeutic option, especially in extravasation injuries involving functionally critical regions that do not respond adequately to conventional approaches.

## Introduction

Vincristine is a vinca alkaloid commonly used in the treatment of pediatric solid tumors and hematologic malignancies (1, 2). Due to its well-known vesicant properties, extravasation may lead to severe soft tissue injury beginning with localized pain and erythema and progressing to necrosis and ulceration (3–5). Particularly in pediatric oncology patients requiring prolonged treatment, this complication may significantly complicate the treatment course.

Several factors increase the risk of extravasation in pediatric patients, including fragile peripheral veins, repeated peripheral venous access, and limited patient cooperation during infusion procedures (6, 7). The standard management of vinca alkaloid extravasation includes immediate cessation of infusion, aspiration of the infiltrated agent whenever possible, elevation of the affected extremity, and warm compress application (3, 4). However, in cases involving deeper tissue injury, these measures may not always be sufficient in preventing progressive tissue damage (5, 6).

Amniotic membrane has long been used as a biological dressing in various wound types due to its anti-inflammatory, antifibrotic, and epithelialization-promoting properties. It is rich in various growth factors such as transforming growth factor beta (TGF-β), epidermal growth factor (EGF), and fibroblast growth factor (FGF), along with essential cytokines which collectively facilitate scarless tissue healing (8–10). Although reports regarding its use in chemotherapy extravasation injuries remain limited, it has been considered a potential therapeutic option in selected cases refractory to standard treatment (9–11). The number of publications in the literature regarding the application of amniotic membrane grafting in chemotherapy-induced extravasation injuries in children is limited. In this report, we describe the case of a pediatric patient with Wilms tumor who developed progressive tissue injury following vincristine extravasation on the dorsolateral region of the foot and was successfully treated with an amniotic membrane graft.

## Case presentation

**Figure 1:**
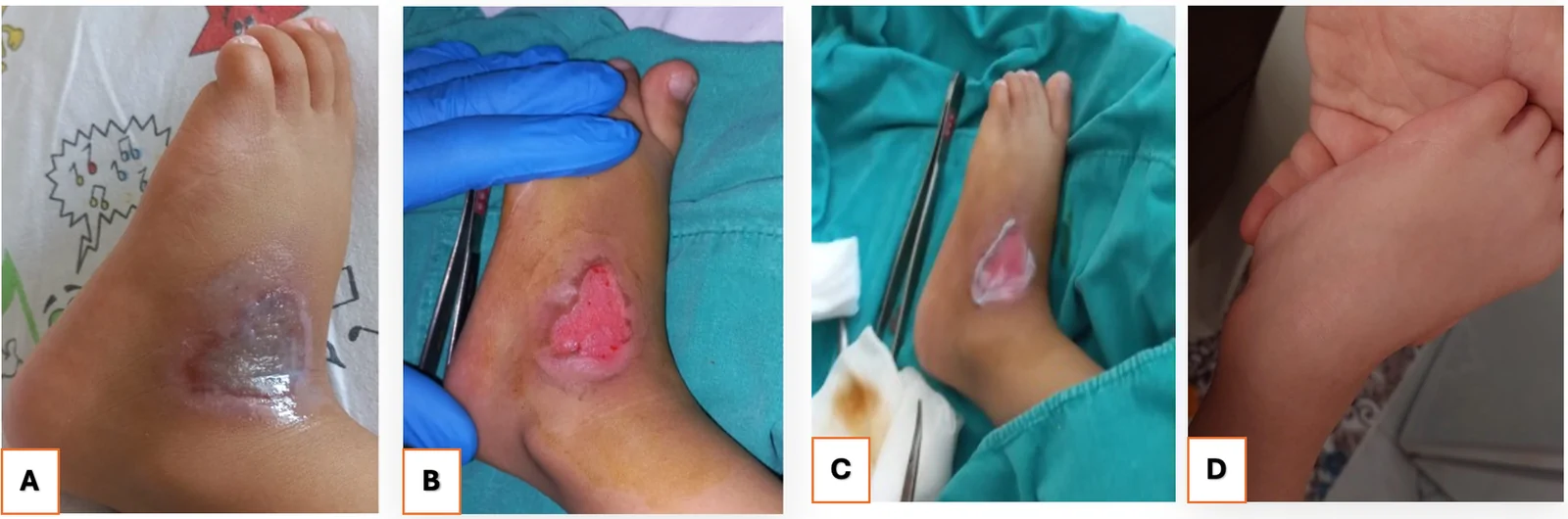
**(A)** Necrotic appearance of the dorsolateral foot region following vincristine extravasation. **(B–D)** Stages of wound healing during amniotic membrane treatment.

A 2-year-8-month-old boy was admitted to our clinic with abdominal swelling and firmness. Physical examination revealed a firm palpable abdominal mass measuring approximately 5 cm × 4 cm in the left abdominal quadrant at the level of the umbilicus. Other system examinations were within normal limits. Following surgical intervention, histopathological evaluation confirmed the diagnosis of nephroblastoma (Wilms tumor). No anaplasia was identified. The disease was classified as Stage II Wilms tumor, and postoperative chemotherapy was initiated. The patient received a chemotherapy protocol including carboplatin, vincristine, and actinomycin-D (12). During week 24 of chemotherapy, vincristine extravasation developed from a peripheral intravenous line located on the dorsolateral aspect of the left foot. Once extravasation was recognized, the infusion was immediately discontinued, aspiration of the infiltrated agent was attempted, the affected extremity was elevated, and warm compress therapy was initiated. The laboratory evaluation at that time showed hemoglobin 12.2 g/dL, platelet count 265 × 10^3^/mm^3^, WBC 5.13 × 10^3^/mm^3^, neutrophil 1.4 × 10^3^/mm^3^, lymphocyte 3.01 × 10^3^/mm^3^, CRP 9.8 mg/L (range 0–5 mg/L). The patient had mild neutropenia at the time of injury and received prophylactic antibiotic therapy. Although the lesion initially appeared limited, progressive deterioration in skin integrity and necrotic tissue formation were observed during the first week of follow-up (Figure 1). The lesion consisted of a well-demarcated necrotic ulcer located on the dorsolateral aspect of the left foot, measuring approximately 3 cm × 2.5 cm. The wound demonstrated full-thickness skin loss with a central black eschar and surrounding erythematous margins, without visible exposure of tendon or bone. The appearance was consistent with progressive soft-tissue necrosis secondary to vincristine extravasation. The patient was evaluated by the plastic surgery department. Because the lesion involved a weight-bearing and functionally important anatomical region, continuation of conservative treatment alone was considered insufficient and associated with a risk of delayed healing and long-term functional impairment. After multidisciplinary evaluation of the clinical course and extent of tissue injury, surgical debridement followed by amniotic membrane graft application was planned. Following debridement of necrotic tissue, a human-derived amniotic membrane graft was placed over the wound bed, and a breathable dressing was applied. Dressings were changed every 2 days for 10 days, after which the wound was left open to heal secondarily. During follow-up, no signs of infection were observed. Progressive reduction in pain, gradual granulation tissue formation, and epithelialization were noted (Figure 1). Complete epithelialization occurred in 8 weeks, with preservation of full foot function and no contracture at 1-year follow-up. No complications occurred during the 1-year follow-up. As the extravasation occurred during the final cycle of chemotherapy, no treatment delay or modification was required.

### The amniotic membrane procedure

The amniotic membrane used in this case was prepared from placental tissue obtained following appropriate donor screening and informed consent procedures. After transfer of the placenta to the processing center under sterile conditions, samples from the transport solution were evaluated with aerobic and anaerobic cultures for sterility assessment. Within a sterile cabinet environment, the amniotic membrane was mechanically separated from the placenta, and chorionic tissue as well as residual blood components were removed using isotonic solution irrigation. The membrane was subsequently preserved in an antibiotic-containing solution at +4 °C until sterility confirmation was achieved. The processed membranes were then cut into appropriate sizes according to clinical requirements and stored under sterile conditions until graft application.

## Discussion

Vincristine extravasation is infrequent; however, it represents a clinically significant complication in pediatric oncology practice because of its potential to cause severe morbidity (3, 6). Extravasation of vesicant vinca alkaloids may induce local inflammation, cellular injury, and progressive tissue necrosis (4, 5). Such injuries may result not only in pain and ulceration but also in prolonged wound healing and long-term functional impairment in selected patients (5, 7). In pediatric patients, the smaller caliber of peripheral veins, frequent need for venous access, and limited patient cooperation further increase the risk of extravasation (6, 7).

Early recognition and prompt intervention remain the cornerstone of extravasation management. Standard treatment includes immediate discontinuation of infusion, aspiration of the infiltrated drug whenever feasible, elevation of the affected extremity, and warm application for vinca alkaloids (3, 4). However, current literature indicates that lesions initially appearing mild may progress over time to involve deeper soft tissues, and conservative approaches may prove inadequate in some cases (5, 6). As a result, careful monitoring and reassessment of lesion progression are essential during follow-up.

In the standard management of vinca alkaloid extravasations, hyaluronidase is widely discussed as a pharmacological antidote due to its ability to degrade hyaluronic acid in the extracellular matrix, which facilitates the rapid dispersion and systemic absorption of the infiltrated vesicant. However, the therapeutic efficacy of hyaluronidase is highly time-dependent, typically requiring administration within the first 1–24 h of the initial insult before fixed tissue necrosis establishes (11). In our patient, despite immediate conservative countermeasures at the time of injury, the ulceration progressively evolved over the first week into a well-demarcated necrotic lesion. At this chronic and structurally disrupted stage, enzymatic dispersion agents like hyaluronidase no longer offer clinical utility. Instead, the management paradigm must shift toward mechanical debridement and advanced wound coverage.

In progressive extravasation injuries, surgical intervention may become necessary. In recent years, increasing attention has been directed toward biological materials that support wound healing following debridement of necrotic tissue (7, 8). Among these, amniotic membrane has been described as a biological dressing with anti-inflammatory, antifibrotic, antimicrobial, and epithelialization-promoting properties that may facilitate wound healing (8–10). While conventional approaches for debrided pediatric wounds often involve silver-based local dressings, vacuum-assisted closure, or autologous skin grafting, these modalities possess notable drawbacks. Autologous split-thickness skin grafts introduce secondary donor-site morbidity and carry a high risk of long-term scar contracture over thin-skinned, highly mobile joint spaces like the dorsolateral foot (13). In contrast, biological scaffolds like the amniotic membrane provide an active bio-microenvironment rather than serving as a passive barrier dressing. Growth factors and cytokines present within the stromal structure of the amniotic membrane have been shown to modulate inflammatory responses by downregulating pro-inflammatory cytokines (such as IL-1 and TNF-α), support fibroblast proliferation, and enhance epithelial cell migration (9, 10). These characteristics may provide clinical benefit particularly in wounds with impaired tissue integrity and delayed healing.

Although studies evaluating amniotic membrane use in pediatric patients remain limited, previous reports have demonstrated accelerated granulation tissue formation and enhanced epithelialization in wounds associated with extravasation injuries from medications and parenteral nutrition products in infants and children (8, 11). In a clinical pilot study, Kadivar et al. demonstrated that using amniotic membrane as a biological dressing for wounds secondary to extravasation in pediatric patients significantly shortened the healing period to an average of 2.5 weeks and resulted in scarless tissue recovery without any adverse reactions (8). However, the literature regarding its use in chemotherapy-induced extravasation injuries is quite limited. Evaluating alternative treatment strategies may be beneficial, particularly in cases resistant to standard treatment and involving functionally critical anatomical regions. In our patient, the lesion was located on the dorsolateral side of the foot, an area subjected to repetitive mechanical stress during walking. For this reason, preserving tissue integrity and preventing contracture were crucial considerations when choosing a reconstructive approach. In the present case, despite initial conservative management, progressive tissue injury and skin breakdown necessitated a multidisciplinary treatment approach. Following evaluation by the plastic surgery team, debridement of necrotic tissue and application of an amniotic membrane graft were performed. Absence of infection, progressive granulation tissue formation, and successful epithelialization after graft application suggest a potentially beneficial role of amniotic membrane in promoting wound healing in chemotherapy extravasation injuries.

The importance of a multidisciplinary approach in the management of chemotherapy-induced extravasation injuries has been highlighted many times in the literature (6, 7). Early recognition of these complications and timely consideration of surgical treatment options when indicated are essential for preservation of long-term functional outcomes in pediatric patients. Last of all, biological materials such as amniotic membrane grafts may represent a promising therapeutic option in selected cases characterized by progressive tissue injury despite conservative management (14).

## Limitations

This report possesses certain limitations inherent to single-case clinical documentation. As an isolated case study without a matched control comparator, it is impossible to definitively separate the independent therapeutic effect of the amniotic membrane graft from the natural biological healing trajectory of the pediatric patient. Furthermore, surgical debridement itself is a well-established driver of wound healing that effectively removes cellular bioburden and necrotic debris, making it a major confounding contributor to the positive outcome observed.

Additionally, prospective planimetric wound-size measurements and validated pediatric pain assessment scores (e.g., the FLACC scale) were not available. Although approximate wound dimensions and healing time were documented retrospectively from the clinical record and serial photographs, clinical priority during this emergency intervention in a highly distressed 2-year-old child was directed toward rapid stabilization and treatment rather than prospective research-oriented documentation. Nevertheless, serial clinical photography (Figure 1) provided visual verification of accelerated tissue reconstruction, and the complete preservation of joint mobility without contracture at the 1-year follow-up offers a strong, clinically meaningful objective endpoint. While these limitations preclude broad causal conclusions, this case provides valuable proof-of-concept data for amniotic membrane grafting as a safe secondary reconstructive modality for progressive pediatric chemotherapy extravasations.

## Conclusion

While early intervention remains the primary approach in the management of vincristine extravasation injuries, surgical treatment and biological wound dressings can provide successful clinical outcomes in selected cases with progressive necrosis. The limited number of reports regarding amniotic membrane use in vincristine extravasation injuries suggests that this case may contribute valuable clinical experience to the literature. Vincristine extravasation is a rare but clinically important complication in pediatric patients. In cases involving progressive tissue injury despite conservative management, particularly in functionally significant regions, amniotic membrane grafting may be considered a supportive therapeutic option that promotes tissue healing and helps preserve functional integrity.

## Data Availability

The original contributions presented in the study are included in the article/Supplementary Material, further inquiries can be directed to the corresponding author.
